# Elevated Atmospheric CO_2_ Concentration Improved C_4_ Xero-Halophyte *Kochia prostrata* Physiological Performance under Saline Conditions

**DOI:** 10.3390/plants10030491

**Published:** 2021-03-05

**Authors:** Zulfira Rakhmankulova, Elena Shuyskaya, Kristina Toderich, Pavel Voronin

**Affiliations:** 1 K.A. Timiryazev Institute of Plant Physiology RAS, IPP RAS, 35 Botanicheskaya St., 127276 Moscow, Russia; zulfirar@mail.ru (Z.R.); pavel@ippras.ru (P.V.); 2 International Platform for Dryland Research and Education, Tottori University, 1390 Hamasaka, Tottori 680-0001, Japan; ktoderich@yahoo.com; 3 International Center for Biosaline Agriculture for Central Asia and Caucasus, Tashkent 100000, Uzbekistan

**Keywords:** elevated CO_2_, saline stress, droughts, combined stress, photosynthesis, photosystems I and II, respiration, proline

## Abstract

A significant increase in atmospheric CO_2_ concentration and associated climate aridization and soil salinity are factors affecting the growth, development, productivity, and stress responses of plants. In this study, the effect of ambient (400 ppm) and elevated (800 ppm) CO_2_ concentrations were evaluated on the C_4_ xero-halophyte *Kochia prostrata* treated with moderate salinity (200 mM NaCl) and polyethylene glycol (PEG)-induced osmotic stress. Our results indicated that plants grown at elevated CO_2_ concentration had different responses to osmotic stress and salinity. The synergistic effect of elevated CO_2_ and osmotic stress increased proline accumulation, but elevated CO_2_ did not mitigate the negative effects of osmotic stress on dark respiration intensity and photosystem II (PSII) efficiency. This indicates a stressful state, which is accompanied by a decrease in the efficiency of light reactions of photosynthesis and significant dissipative respiratory losses, thereby resulting in growth inhibition. Plants grown at elevated CO_2_ concentration and salinity showed high Na^+^ and proline contents, high water-use efficiency and time required to reach the maximum P700 oxidation level (PSI), and low dark respiration. Maintaining stable water balance, the efficient functioning of cyclic transport of PSI, and the reduction of dissipation costs contributed to an increase in dry shoot biomass (2-fold, compared with salinity at 400 ppm CO_2_). The obtained experimental data and PCA showed that elevated CO_2_ concentration improved the physiological parameters of *K. prostrata* under salinity.

## 1. Introduction

According to the Intergovernmental Panel on Climate Change (IPCC), a significant increase in atmospheric CO_2_ concentration, associated climate aridization, and soil salinity are factors affecting the growth, development, and functioning of plants, which can potentially change the composition of plant communities, spread of ecosystems, and lead to a catastrophic decrease in biodiversity [1,2,3]. Therefore, issues related to the impact of global climatic changes on vegetation are leading in biological science. C_4_ plants have long been considered less dependent on environmental CO_2_ concentration than their C_3_ counterparts due to the presence of a carbon-concentrating mechanism (CCM), which makes them less responsive to an increase in atmospheric CO_2_ concentration [4,5]. This concept is deeply embedded in climatic and ecological models of climate change impact on plants [5,6,7]. However, recently, contradicting information appeared on this issue. On the one hand, it was shown that in C_4_ halophytes of the Chenopodiaceae family, a high CO_2_ concentration more effectively stimulates photosynthetic metabolism than in C_3_ species [8] and is associated with an improved water-use efficiency (WUE) [2]. Alternatively, high CO_2_ concentration may not lead to significant changes in the visible photosynthesis and transpiration [2] and may even decrease the carboxylation efficiency and the CO_2_ saturated rate of photosynthesis in C_4_ plants [9]. For example, it has been shown that high (>800 ppm) and ultra-high (>1000 ppm) CO_2_ concentrations decrease the intensity of photosynthesis in both C_3_ and C_4_ species [3,10]. Based on this, experiments that studied the effect of “doubling the CO_2_ concentration” in C_4_ plants are relevant, since modern climate models can both overestimate or underestimate the potential impact of future climate changes on global agriculture and ecosystems [3]. Conditions accompanying an increase in CO_2_ concentration in the atmosphere, such as intense sunlight, drought, variable and elevated temperatures, salinity, and the availability of essential nutrients can become inhibitory factors for plant growth [11]. Water deficit and salinity, which are considered more relevant within a 30-year time frame will have a stronger negative effect on the planet survival than the actual change in CO_2_ atmospheric concentration [11]. Studies on the synergistic effect of high CO_2_ concentration and moderate drought have shown positive effects of high CO_2_ conditions on the drought tolerance of some C_3_ and C_4_ species [12,13]. However, according to some studies, C_3_ plants are more competitive than C_4_ species under these conditions [14,15], while according to others, the intensity of photosynthesis increases in C_4_ plants [15,16] due to more efficient water usage [17]. A study on the combined effect of high CO_2_ concentration and salinity showed that increased CO_2_ concentration (500 ppm) enhances the expression of salt-sensitive genes in both C_3_ and C_4_ species [8]. In the C_3_ halophyte *Salicornia ramosissima*, the synergistic effect of 700 ppm CO_2_ at 510 mM NaCl led to a high net photosynthesis rate and an improvement in the water balance of plants associated with a decrease in stomatal conductance, and adjustment of osmotic potential due to an increase in Na^+^ content in plant tissues [1], which resulted in increased WUE [18]. Despite these positive effects, no significant changes in biomass growth were observed. The authors explained this fact as the investment of the higher energy in protective mechanisms against salt stress [1].

Due to the multifactorial nature of global climatic changes, species characterized by drought and salt tolerance, xero-halophytes, are of increasing interest. These plants possess a complex of protective and adaptive strategies that allow them to resist and actively grow under conditions of osmotic stress, ionic toxicity, impaired mineral nutrition and metabolism, oxidative stress, disorganized membranes, and to use the energy potential more efficiently [19]. Therefore, xero-halophytes serve as an important resource for studying the mechanisms of drought and salt tolerance as well as identifying and developing new systems in crop production and phytomelioration [20,21]. One of the largest families of the arid and semi-arid territories is the Chenopodiaceae, a feature of which is the presence of closely related species—xero-halophytes—with different types of photosynthetic metabolism and different tolerance to drought and salinity [22]. Currently, species of this family are used to study C_4_ evolution [23,24], and they are also of practical interest; particularly, they are also actively used in ecological restoration of degraded agricultural landscapes and marginal territories in the production of forage in dry lands [25]. Studies on the impact of high CO_2_ concentration on the tolerance of xero-halophytes to drought and salinity are insufficient and fragmentary. This study compared the effect of ambient (400 ppm) and elevated CO_2_ concentration (800 ppm) on the tolerance of the C_4_ xero-halophyte *Kochia prostrata* (Chenopodiaceae) to moderate salinity (200 mM NaCl) and polyethylene glycol (PEG)-induced osmotic stress with a similar osmotic potential (−0.6 MPa).

## 2. Results

**Figure 1:**
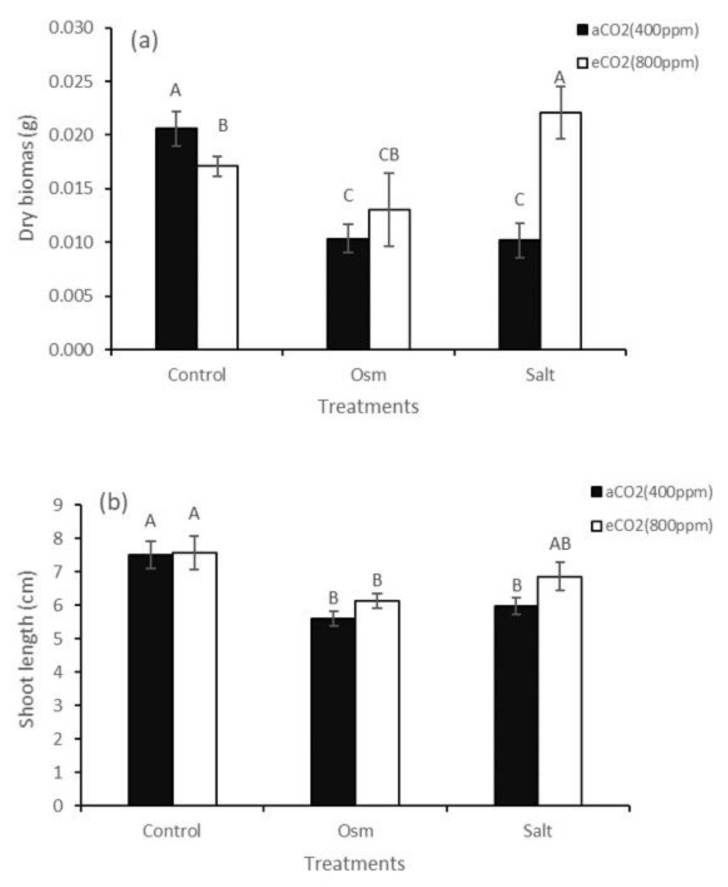
Dry biomass (**a**) and shoot length (**b**) of C_4_-species *Kochia prostrata* at different CO_2_ concentrations (400 and 800 ppm) under moderate (–0.6 MPa) polyethylene glycol (PEG)-induced osmotic stress (Osm) and salinity (Salt) similar in osmotic potential (200 mM NaCl). The values are the means (±SE) of seven replicates. Different letters above the bars represent significant differences at the *p* < 0.05 (Tukey’s pairwise comparison). aCO_2_—ambient CO_2_ concentration; eCO_2_—elevated CO_2_ concentration.

The C_4_ xero-halophyte *Kochia prostrata* grown at an ambient CO_2_ concentration (aCO_2_) was equally intolerant to moderate osmotic stress and salinity. Dry biomass and shoot length decreased 2-fold and 1.3-fold, respectively (Figure 1a,b). Growing plants under elevated CO_2_ (eCO_2_) led to a slight decrease in dry biomass accumulation in control plants (1.2-fold) (Figure 1a), but the shoot length remained unchanged (Figure 1b). At elevated CO_2_, a 4-d exposure to osmotic stress (eCO_2_ + Osm) did not affect dry biomass and reduced the shoot length of plants (1.2-fold) (Figure 1a,b). Whereas, a 4-d salinity exposure at elevated CO_2_ (eCO_2_ + Salt) stimulated dry biomass accumulation by 2-fold (compared with aCO_2_ + Salt) (Figure 1a).

**Figure 2:**
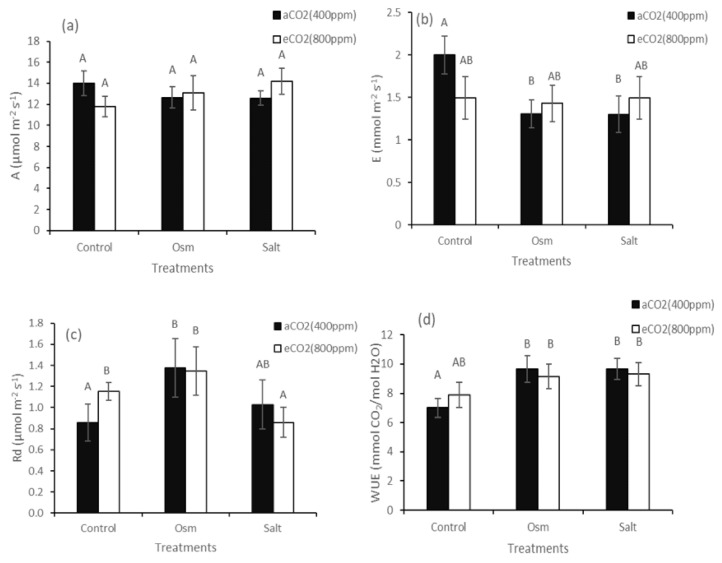
Apparent photosynthesis (**a**), transpiration (**b**), dark respiration (**c**), and water-use efficiency (**d**) in plant leaves of C_4_ *Kochia prostrata* at different CO_2_ concentrations (400 and 800 ppm) under moderate (–0.6 MPa) PEG-induced osmotic stress (Osm) and salinity (Salt) similar in osmotic potential (200 mM NaCl). The values are means (±SE) of seven replicates. Different letters above the bars represent significant differences at the *p* < 0.05 (Tukey’s pairwise comparison). aCO_2_—ambient CO_2_ concentration; eCO_2_—elevated CO_2_ concentration.

A study on CO_2_/H_2_O gas exchange in *K. prostrata* showed that the intensity of apparent photosynthesis (A) did not significantly change at elevated CO_2_ as well as at PEG-induced osmotic stress and moderate salinity with a similar osmotic potential (Figure 2a). Transpiration intensity (E) decreased 1.4-fold in plants at both aCO_2_ + Osm and aCO_2_ + Salt. Elevated CO_2_ did not affect the transpiration intensity at all treatments (Figure 2b). Dark respiration intensity (Rd) increased 1.5-fold at aCO_2_ + Osm, 1.3-fold at eCO_2_, and 1.5-fold under the combined effect of osmotic stress and elevated CO_2_ (eCO_2_ + Osm) (Figure 2c). Under eCO_2_ + Salt, there was a decrease in Rd to control values at 400 ppm (aCO_2_). A decrease in transpiration intensity led to a 1.4-fold increase in water-use efficiency at all treatments (Figure 2d).

**Figure 3:**
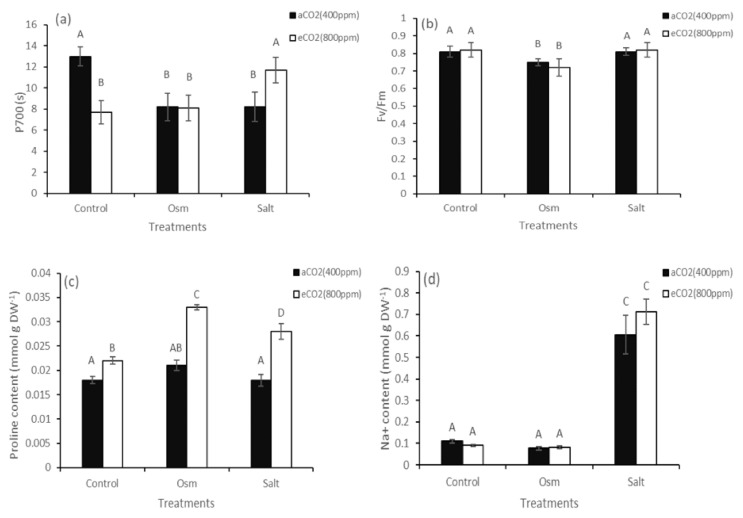
Time required to reach the maximum P700 oxidation level under far-red light (PSI) (**a**), maximum quantum yield of PSII reaction (**b**), content of free proline (**c**) and content of Na^+^ (**d**) in plant leaves of C_4_ *Kochia prostrata* at different CO_2_ concentrations (400 and 800 ppm) under moderate (−0.6 MPa) PEG-induced osmotic stress (Osm) and salinity (Salt) similar in osmotic potential (200 mM NaCl). The values are means (±SE) of seven replicates. Different letters above the bars represent significant differences at the *p* < 0.05 (Tukey’s pairwise comparison). aCO_2_—ambient CO_2_ concentration; eCO_2_—elevated CO_2_ concentration.

A study on the efficiency of photosystems of *K. prostrata* showed that the time required to reach the maximum P700 oxidation level under far-red light (PSI) decreased 1.6-fold at both aCO_2_ + Osm and aCO_2_ + Salt. In plants grown under eCO_2_, this parameter decreased 1.7-fold (Figure 3a). Similar changes were seen in plants grown under elevated CO_2_ and exposed to osmotic stress (eCO_2_ + Osm). However, at eCO_2_ + Salt, it was observed that the time required to reach the maximum P700 oxidation level increased to values at aCO_2_ (Figure 3a). PSII efficiency decreased at aCO_2_ + Osm and under the combined effect of eCO_2_ + Osm (Figure 3b).

A study on free proline content showed a 1.2-fold increase in plants at eCO_2_. The combined effect of eCO_2_ + Salt led to a 1.3-fold increase in proline content. The most significant increase (1.5-fold) in proline content was observed under the combined effect of eCO_2_ + Osm (Figure 3c). At both aCO_2_ + Salt and eCO_2_ + Salt, an increase in Na^+^ accumulation in *K. prostrata* tissues was observed (on average, 6-fold higher than in control plants) (Figure 3d). There were no differences in K^+^ content and K^+^/Na^+^ ration under stress conditions at both CO_2_ concentrations. K^+^ content varied from 1.48 ± 0.31 to 1.76 ± 0.03 mmol g^−1^ DW under stress conditions regardless of CO_2_ concentrations. K^+^/Na^+^ ration averaged 19.3 ± 0.9 under osmotic stress and 2.7 ± 0.2 under salinity at both CO_2_ concentrations.

**Figure 4:**
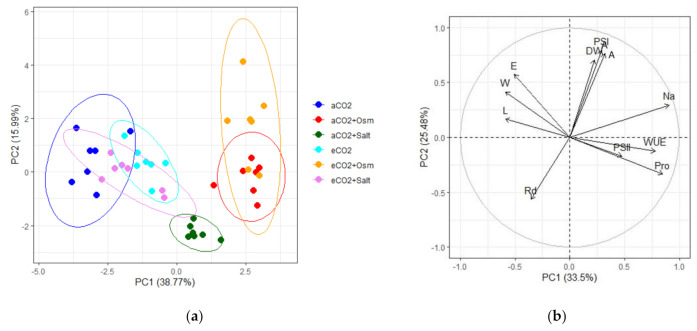
Principle component analysis (PCA) (**a**) score plot of the physiological data of C_4_ *Kochia prostrata* at different CO_2_ concentrations (400 and 800 ppm) under moderate (–0.6 MPa) PEG-induced osmotic stress and salinity similar in osmotic potential (200 mM NaCl) and (**b**) multiple correlation of the physiological data of C_4_ *Kochia prostrata*. aCO_2_—ambient (400 ppm) CO_2_ concentration without treatment; aCO_2_ + Osm—ambient CO_2_ + PEG-induced osmotic stress; aCO_2_ + Salt—ambient CO_2_ + NaCl-induced salinity; eCO_2_—elevated (800 ppm) CO_2_ concentration; eCO_2_ + Osm—elevated CO_2_ + PEG-induced osmotic stress; eCO_2_ + Salt—elevated CO_2_ + NaCl-induced salinity. Parameters abbreviations are listed in table.

To assess the possible synchronous impact of eCO_2_ + Salt and eCO_2_ + Osm on the growth and physiological reactions of *K. prostrata*, principal component analysis (PCA) of biochemical and physiological parameters was performed. Plants grown at ambient CO_2_ were distinguished into three groups: control, drought, and saline conditions, separated by the first main component (PC1) (Figure 4a). Within plants grown at elevated CO_2_, groups of plants under osmotic stress (eCO_2_ + Osm) were distinguished by PC1 and intersected with plants grown at aCO_2_ + Osm (Figure 4a). Plants at eCO_2_ + Salt overlapped with those at both aCO_2_ and eCO_2_. The main elements of PC1 were growth parameters: shoot length (L) and dry biomass (DW) as well as PSII efficiency (Table 1). The second main component (PC2) did not give a clear division into groups, except for aCO_2_ + Salt. Here, the main significant factors were water, Na^+^, and proline content. The first two principal components (PC1 and PC2) are sufficient to explain 55% of the total variation.

To identify the relationships between factors involved in the adaptive mechanisms to salinity at elevated CO_2_ conditions, a multivariate statistical approach using PCA was performed (Figure 4b). The results revealed that the aboveground dry biomass positively correlated with the apparent photosynthesis intensity (A) and the time required to reach the maximum P700 oxidation level under far-red light (PSI), i.e., with the activity of cyclic electron transport. These parameters negatively correlated with the dark respiration intensity (Rd). Na^+^ accumulation was not directly related to plant productivity. Na^+^ and proline contents correlated positively with WUE and maximum quantum yield of PSII, i.e., maintaining water balance, ensuring high WUE, and efficiency of the photosynthetic electron transport chain.

## 3. Discussion

The negative effect of salinity on plant growth is associated with a low osmotic potential of the soil solution (osmotic stress) and toxic ionic effects (ionic stress); therefore, in this study, we investigated the differential effects of elevated CO_2_ concentration on the tolerance of C_4_ xero-halophyte *K. prostrata* to PEG-induced osmotic stress and NaCl-induced salinity (combined ionic and osmotic stress). Both of these stresses are usually accompanied by oxidative stress in plants. In our experiments, we specially used low PEG and NaCl concentrations for euhalophyte to create moderate osmotic and ionic stresses, which do not cause significant damage to photosynthetic membranes (PS II, Figure 3b) and cytotoxic effect (K^+^ and proline contents did not change under both stresses at 400 ppm CO_2_). Halophytes adapt to salinity, accumulating Na^+^ ions through selective ion transport and ionic compartmentation (usually in vacuoles) [26], while they synthesize compatible solutes in the cytoplasm to prevent adverse effects of salts on metabolism and growth [27]. Growth parameters are an integral characteristic of the implementation of adaptive physiological and biochemical mechanisms. Plants grown at elevated CO_2_ (800 ppm) showed a decrease in dry biomass compared with control plants at 400 ppm CO_2_ (Figure 1). Although there is ample evidence that C_4_ plants can accumulate more biomass at elevated CO_2_ partial pressure, the mechanisms underlying this response are largely unclear [28]. Elevated CO_2_ can influence the biomass accumulation of C_4_ plants in two ways: due to an increase in the rate of CO_2_ assimilation in leaves because of an increase in intercellular CO_2_ concentration or through a decrease in stomatal conductance and, accordingly, the rate of leaf transpiration. A decrease in the transpiration rate can improve water exchange in shoots and increase leaf temperature [26]. In addition to these two main components, the intensity of dark respiration can also affect dry biomass accumulation [15,29], because Rd can significantly increase with an increase in leaf temperature [28]. Understanding the role of plant respiration at elevated CO_2_ is further complicated by the presence of an alternative pathway, which consumes photosynthetic products without producing chemical energy (ATP) [30], thereby leading to significant dissipation losses. Analysis of CO_2_/H_2_O gas exchange in the C_4_ xero-halophyte *K. prostrata* did not reveal significant changes in apparent photosynthesis and transpiration intensity and, accordingly, WUE at elevated CO_2_ concentration (Figure 2a,b,d). A decrease in biomass under these conditions can be explained by an increase in the dark respiration intensity (Figure 2c), which is possibly associated with additional energy costs for proline biosynthesis [31], leading to a 1.2-fold increase in its content (Figure 3c), also with an increase in dissipation processes.

In C_4_ plants, both C_3_ and C_4_ photosynthesis cycles are functionally active, increasing the energy cost of CO_2_ assimilation in comparison with C_3_ plants. Consequently, two additional ATP molecules are required for each CO_2_ molecule fixed by the C_4_ cycle. It is assumed that additional ATP is produced by cyclic electron transport around PSI, contributing to the generation of a pH gradient across the thylakoid membrane without the formation of nicotinamide adenine dinucleotide phosphate (NADPH) [32]. Comparative analysis within the genus, *Flaveria*, which includes C_3_, intermediate C_3_–C_4_, and C_4_ species showed that C_4_ plants exhibited higher gene expression of proteins involved in the cyclic electron transport of PSI and changes in the thylakoid structure, thereby contributing to an increased activity of the cyclic electron flux [32]. In our experiments, *K. prostrata* grown at elevated CO_2_ conditions showed a 1.7-fold decrease in the time required to reach the maximum P700 oxidation level under far-red light (PSI), i.e., a decrease in the intensity of cyclic electron transport, which indirectly indicates a decrease in the activity of C_4_ CCM. Thus, a decrease in plant dry biomass at elevated CO_2_ can be associated with both an increase in dissipation costs during respiration and a decrease in the intensity of the cyclic electron transport around PSI and, possibly, with a less efficient CCM.

Conditions that accompany an increase in atmospheric CO_2_ also have a significant impact on other environmental factors. Particularly, water deficit and salinity, separately or interacting with each other, can inhibit plant growth [11]. Our studies have shown that at ambient CO_2_, the C_4_ xero-halophyte *K. prostrata* is intolerant to moderate salinity (200 mM NaCl) and moderate PEG-induced osmotic stress with a similar osmotic potential (−0.6 MPa). A significant decrease in dry biomass accumulation and plant height was observed at both treatments (Figure 1a,b). *K. prostrata* grown under elevated CO_2_ reacted differently to osmotic stress and salinity (osmotic and ionic stress). At elevated CO_2_, plant dry biomass increased (up to aCO_2_) only under salinity. Under these conditions, elevated Na^+^ and proline contents in plant tissues were observed (Figure 3c,d). Sodium ions are cheap osmoticum to lower the cell osmotic potential and hence prevent water loss [26]. In the experiments, we used moderate salinity (200 мM NaCl), which caused 2-fold decreased dry biomass, but it did not lead to cytotoxicity, since a noticeable kosmotropic effect of Na^+^ in halophytes usually requires a concentration >200 мM NaCl [26]. The amino acid proline plays an important role in plant metabolism and development. Free proline participates in the maintenance of cellular homeostasis, including redox balance and energy status, and it acts as an osmolyte and antioxidant under stress conditions. Proline can function as a signaling molecule [31]. In C_4_ halophytes (*Suaeda monoica, S. fruticosa*) grown at high CO_2_ concentration (900 ppm), 13-54-fold increased proline content was shown as compared to plants grown at 400 ppm CO_2_ [33]. Under high CO_2_ concentration, free proline can play an osmoprotective, regulatory, antioxidant, and energetic function in C_4_ halophytes. The sodium and proline accumulation ensured a stable water balance in *K. prostrata* plants, which is confirmed by the absence of a decrease in the maximum quantum yield of PSII (Figure 3b), constant A and E (Figure 2a,b), a decrease in dissipation costs Rd (Figure 2c), and a 1.4-fold increase in the time required to reach the maximum P700 oxidation level (PSI) (compared with aCO_2_ + Salt) (Figure 3a), and, probably, the activation of cyclic electron transport intensity led to a 2-fold increase in dry biomass (compare with aCO_2_ + Salt) (Figure 1a) up to the growth of plants at aCO_2_. The positive effect of elevated CO_2_ concentration on salt tolerance was seen in some C_4_ halophytes: an increase in salt-responsive genes expression [8] and in WUE [1,18].

The combine effect of elevated CO_2_ concentration and osmotic stress led to high proline content and dark respiration intensity (Figure 2c and Figure 3c), indicating a stress state, de-coordination of physiological processes, and an increase in dissipation costs. A decrease in PSII efficiency, as well as the reduced time required to reach the maximum P700 oxidation level (PSI) (Figure 3a,b) indicates a deficit in energy, which decreased growth parameters (compare with aCO_2_). However, note that if a decrease in dry biomass was significant at aCO_2_ + Osm (2-fold), then at eCO_2_ + Osm, a decrease in growth is insignificant (Figure 1a). Thus, elevated CO_2_ has some softening and protective effect on osmotic stress, which is consistent with the results in other studies [12,13].

## 4. Materials and Methods

### 4.1. Plant Material

*Kochia prostrata* [L.] Schrad. (Chenopodiaceae) is a highly productive, drought- and salt-tolerant plant grown in arid and semi-arid rangelands of Central Eurasia and the western U.S. [34,35,36]. *K. prostrata* is typical salt accumulating halophyte whose optimal germination occurred at 0–1% NaCl [37] and optimal growth occurred up to 150 mM NaCl [38]. *K. prostrata* also has great potential for establishing palatable perennial shrubs in arid rangeland at 70 mm annual precipitation [39] and in saline soils (EC = 20 dS/m) [36].

### 4.2. Growth Conditions

Seeds of *Kochia prostrata* (L.) Schrad. were germinated on filter paper soaked in distilled water within 7–11 days. After that, the seedlings were transplanted to perlite in plastics containers of 24-cm length, 20-cm width, and 10-cm depth. There were 20 seedlings per container. Each plastic container was placed on separate plastic tray. During next 30 days, the seedlings were grown using the nutrient solution 50% Hoagland, which was added to each plastic tray. The seedlings were grown in two separated climate chambers under circadian illumination (using commercial luminescent white light tubes): 10-h dark/14-h light (200 µmol m^−2^ s^−1^ PAR, light meter LI-205A (Li-Cor, USA)), 25 ± 5 °C temperature and two levels of CO_2_ concentrations: in the first chamber, 60 plants were grown at ambient (400 ppm) CO_2_, and in the second chamber, another 60 plants were grown at elevated (800 ppm) CO_2_ [40]. When plants were 30 days old, we started treatment with solutes of 15.8% (m/v) PEG 6000 (−0.6 MPa) (20 plants at 400 ppm and 20 plants at 800 ppm CO_2_) and 200 mM NaCl with equivalent osmotic potential (20 plants at 400 ppm and 20 plants at 800 ppm CO_2_). The osmotic potential of the experimental solution was measured using a freezing-point osmometer Osmomat 030 (Gonotec, Germany). Experimental solutions of PEG and NaCl were prepared on the basis of 50% Hoagland solution. A total of 20 plants at 400 ppm and 20 plants at 800 ppm CO_2_ continued to grow on 50% Hoagland in solution and then were used as control. Solutions were added to a plastic tray. Plants were treated by PEG and NaCl during 4 days. The physiological measurements were carried out on the plants at the end of the 4th day of treatment. In general, 6 experimental variants were used: (1) growing at ambient (400 ppm) CO_2_ without treatment (aCO_2_); (2) growing at 400 ppm CO_2_ + 4 days treated by PEG-induced osmotic stress (aCO_2_ + Osm); (3) growing at 400 ppm CO_2_ + 4 days treated by NaCl-induced salinity (aCO_2_ + Salt); (4) growing at elevated (800 ppm) CO_2_ without treatment (eCO_2_); (5) growing at 800 ppm CO_2_ + 4 days treated by PEG-induced osmotic stress (eCO_2_ + Osm); (6) growing at 800 ppm CO_2_ + 4 days treated by NaCl-induced salinity (eCO_2_ + Salt). Plant tolerance to osmotic stress/salinity was assessed by the decrease in its productivity compared with the control.

### 4.3. Dry Biomass and Water Content

At the end of the experiment, water content (W, g g^−1^ DW) was assessed for the shoots in all the groups. Biomass was estimated for dry shoots (DW). Plant samples were dried at 80 °C for 2 days until reaching a constant mass in order to measure quantitatively the dry shoot matter. The water content in the shoots for each treatment and control group was calculated as W = (FW − DW)/DW.

### 4.4. Proline and Na^+^ Ion Contents

Free proline was determined according to Bates [41] with modifications. Dry shoot samples (0.2 g) from each group were homogenized in 2 mL of boiling distilled water, heated at 100 °C for 10 min in a water bath, and then, the homogenates were centrifuged (5 min, 14,000 g). One ml of homogenate was reacted with one ml of acidic ninhydrin (ninhydrin 1% (*w*/*v*) in acetic acid 60% (*v*/*v*), ethanol 20% (*v*/*v*)), and one ml of glacial acetic acid in a tube for 1 h at 100 °C in a water bath, and the reaction was terminated in an ice bath. The mixtures were read at 520 nm using a spectrophotometer (Genesys 10 UV Scanning, ThermoFisher Scientific, USA). Proline concentrations were determined using a calibration curve and expressed as mg g^−1^ DW. Na^+^ and K^+^ contents in the shoots were determined in water extracts from 100 mg of dry samples by atomic absorption spectrometry (Hitachi 207, Hitachi, Tokyo, Japan), according to the manufacturer’s standard protocol.

### 4.5. CO_2_/H_2_O Gas Exchange

The CO_2_/H_2_O exchange was analyzed by placing a leaf segment into a temperature-controlled leaf chamber where the sample was illuminated through a fiber-optic light guide from a KL 1500LCD light source (Schott, Germany). The steady-state CO_2_/H_2_O exchange rates at the leaf–air interface were measured with a single-channel LI-820 infrared gas analyzer (LI-COR, United States) in the open-circuit mode. Apparent photosynthesis (A) was expressed as µmol (CO_2_) m^−2^ s^−1^. The leaf transpiration (*E*, mmol (H_2_O) m^−2^ s^−1^) was calculated from the difference in gas humidity at the inlet and outlet from the leaf chamber. In this experimental system, the humidity of gas flow at the entrance to the leaf chamber was kept constant at a known level using a LI-610 dew point generator (LI-COR). Humidity at the exit of the leaf chamber was determined with a HMP50 psychrometric sensor (Vaisala INTERCAP, Finland). Water-use efficiency (WUE) was calculated as the ratio of apparent photosynthetic assimilation to the transpiration rate (*A*/*E*). After CO_2_/H_2_O gas exchange measuring, the light was turned off, and after steady state, the dark respiration (Rd, µmol (CO_2_) m^−2^ s^−1^) was measured.

### 4.6. Photosystem I

The redox potential changes of chlorophyll P700 were measured by monitoring the leaf absorbance at 820 nm using a dual-wavelength ED-P700DW pulse modulated system (Walz, Germany) in combination with a PAM 101 fluorometer (Walz) [42]. The kinetics of P700 oxidation was measured under illumination with far-red light (720 nm, 17.2 W m^−2^). The level of maximum P700 oxidation was determined by applying the flash from a xenon gas-discharge lamp (50 ms, 1500 Wm^−2^; Walz) in the presence of far-red light.

### 4.7. Photosystem II

The quantum yield of PSII photoreaction in dark adapted (20 min) leaf was determined with a pulse-amplitude-modulated chlorophyll fluorometer (PAM 101, Walz) [43]. The ratio of variable to maximum chlorophyll *a* fluorescence (*F*_v_/*F*_m_) was used as a measure of the maximum quantum yield of PSII reaction. During measurements, the sample was illuminated with weak modulated red light. The output signal of PAM 101 was processed with an analog–digital convertor (PDA-100, Walz) and displayed on a computer. The potential photosynthetic efficiency of dark adapted leaves was estimated from the values of minimal (*F*_0_) and maximal (*F*_m_) fluorescence using an expression: *F*_v_/*F*_m_= (*F*_m −_ *F*_0_)/*F*_m_.

### 4.8. Statistical Analysis

All of the physiological measurements were performed seven times, and the means and standard errors (SEs) are calculated using Sigma Plot 12.0 statistical program. Comparisons of parameters were made between treatments using analysis of variance (ANOVA) with the Tukey test. Differences were considered significant at *p* < 0.05. Statistical software package R was used to perform a multivariate statistical approach using a principal component analysis model (PCA).

## 5. Conclusions

It is clear that future climate conditions, such as elevated CO_2_, drought, and salinity, are likely to negatively impact the growth and productivity of plants. Results obtained showed that the C_4_ xero-halophyte *K. prostrata* grown at ambient CO_2_ concentration (400 ppm) was equally intolerant to osmotic stress and salinity (a combination of osmotic and ionic stress). Dry biomass and shoot length decreased under both stresses. A decrease in the time required to reach the maximum P700 oxidation level (PSI) indirectly indicates a decrease in the intensity of cyclic electron transport, which is inherent in C_4_ plants. A decrease in transpiration intensity also increased water-use efficiency. The differences in plant response to osmotic stress and salinity at 400 ppm CO_2_ were as follows: PSII efficiency decreased and dark respiration increased under osmotic stress, while Na^+^ ion accumulation increased in plant tissues under salinity.

At elevated CO_2_, *K. prostrata* reacted differently to osmotic stress and salinity. The synergetic effect of elevated CO_2_ and osmotic stress resulted in a high proline content, but elevated CO_2_ did not mitigate the negative effects of osmotic stress on dark respiration intensity and PSII efficiency. All these indicate a stressful state, which is accompanied by a decrease in the efficiency of light reactions of photosynthesis and significant dissipative respiratory losses, thereby decreasing growth parameters. Plants grown at elevated CO_2_ conditions and treated with salt displayed Na^+^ and proline accumulation in plant tissues, high WUE and time required to reach the maximum P700 oxidation level (1.4-fold, compared with aCO_2_ + Salt), and low dark respiration efficiency. Maintaining a stable water exchange, efficient functioning of the cyclic transport of electrons of PSI, and decrease in dissipation costs probably contributed to an increase in dry shoot biomass (2-fold, compared with aCO_2_ + Salt). Thus, based on the results, we can conclude that elevated CO_2_ concentration has a positive effect on the productivity and tolerance to salinity of the C_4_ xero-halophyte *K. prostrata*, maintaining the water and energy status, and stimulating the cyclic electron transport, producing additional ATP for the C4 carbon-concentrating mechanism. All this contributes to the maintenance of high dry biomass.

## Figures and Tables

**Table 1:** Factor loading of physiological parameters on axes 1 and 2 of the principal component analysis.

Parameters	PC1	PC2
Photosystem II (PSII)	**−0.3219**	−0.3105
Photosystem I (PSI)	−0.2527	−0.1142
Shoot length (L)	**−0.3614**	0.2312
Dry biomass (DW)	**−0.3445**	0.3182
Water content (W)	−0.2508	**−0.3807**
Proline (Pro)	0.1590	**0.3675**
Na^+^ ions (Na)	−0.1063	**−0.3773**
Apparent photosynthesis (A)	−0.1480	0.2795
Transpiration (E)	−0.2643	0.3570
Water-use efficiency (WUE)	0.2540	−0.0673
Dark respiration (Rd)	0.3146	0.2831

The main significant factors are bold.

## Data Availability

The data presented in this study are available on request from the corresponding author.
